# Characterization of *SLC34A2* as a Potential Prognostic Marker of Oncological Diseases

**DOI:** 10.3390/biom11121878

**Published:** 2021-12-14

**Authors:** Ramilia Vlasenkova, Alsina Nurgalieva, Natalia Akberova, Mikhail Bogdanov, Ramziya Kiyamova

**Affiliations:** 1 Department of Biochemistry, Biotechnology and Pharmacology, Institute of Fundamental Medicine and Biology, Kazan Federal University, 420008 Kazan, Russia; r.mukhamadeeva@yandex.ru (R.V.); alsina97@mail.ru (A.N.); nakberova@mail.ru (N.A.); mikhail.v.bogdanov@uth.tmc.edu (M.B.); 2 Department of Biochemistry and Molecular Biology, McGovern Medical School, The University of Texas Health Science Center, Houston, TX 77030, USA

**Keywords:** cancer, *SLC34A2*, NaPi2b, prognostic marker, biomarker

## Abstract

The main goal of this study is to consider *SLC34A2* as a potential prognostic marker of oncological diseases using the mutational, expression, and survival data of cancer studies which are publicly available online. We collected data from four databases (cBioPortal, The Cancer Genome Atlas; cBioPortal, Genie; International Cancer Genome Consortium; ArrayExpress). In total, 111,283 samples were categorized according to 27 tumor locations. Ninety-nine functionally significant missense mutations and twelve functionally significant indel mutations in *SLC34A2* were found. The most frequent mutations were *SLC34A2-ROS1*, p.T154A, p.P506S/R/L, p.G257A/E/R, p.S318W, p.A396T, p.P410L/S/H, p.S461C, p.A473T/V, and p.Y503H/C/F. The upregulation of *SLC34A2* was found in samples of myeloid, bowel, ovarian, and uterine tumors; downregulation was found in tumor samples of breast, liver, lung, and skin cancer tumors. It was found that the life expectancy of breast and thymus cancer patients with an *SLC34A2* mutation is lower, and it was revealed that *SLC34A2* overexpression reduced the life span of patients with brain, ovarian, and pancreatic tumors. Thereby, for these types of oncological diseases, the mutational profile of *SLC34A2* can be a potential prognostic marker for breast and thymus cancers, and the upregulation of *SLC34A2* can be a potential prognostic marker for brain, ovarian, and pancreatic cancers.

## 1. Introduction

The search for and the characterization of new molecular markers of oncological diseases are important for the prediction of the prognosis of the disease and the response of the tumor to chemotherapy. The majority of these tumor-targeting compounds are directed against cell membrane-bound proteins [1]. However, there is surprisingly little knowledge about which targets should be used for optimal results per the tumor type, or even better, per the individual tumor or patient. Cell membrane transporter proteins such as transporters belonging to glucose transporter GLUT, ATP-binding cassette transporter ABC, and solute carrier transporter SLC families are upregulated on cancer cells, compared to adjacent normal cells. High levels of transporters are found in a wide range of solid tumors, correlating with poor survival [1]. One of the potential molecular tumor markers may be the sodium-dependent phosphate transporter NaPi2b encoded by the *SLC34A2* gene. *SLC34A2*, belonging to the solute carrier gene family, encodes the type II Na/Pi co-transporter (NaPi2b) [2]. NaPi2b is a multitransmembrane sodium-dependent phosphate transporter responsible for transcellular inorganic phosphate absorption [3]. NaPi2b is highly abundant in the brush-border membrane (BBM) of the small intestine, where it is involved in the transcellular flux of inorganic phosphates via the apical membrane of epithelial cells [4,5]. An altered expression of sodium-dependent phosphate transporter NaPi2b has been reported in ovarian cancer [6], lung cancer [7,8,9,10], gastric cancer [11], thyroid cancer [12], and other cancers [13]. The phosphate transporter NaPi2b was identified as a target for MX35 monoclonal antibodies using modified SEREX (the serological analysis of recombinant cDNA expression libraries) technology [14,15]. Currently, NaPi2b is a target for therapeutic antibodies XMT-1536 and XMT-1592, which are in clinical trials for the treatment of ovarian and lung cancers [16]. Despite the fact that NaPi2B is expressed in normal tissues, therapeutic MX35 and Rebmab200 mAbs [17] are accumulated predominantly in cancer tissues. Therefore, NaPi2b could be considered as a potential molecular marker of several types of cancer, and represents a new family of potential cell-surface targets for the immunotherapy of cancer.

At the moment, there is limited evidence that NaPi2b has potential as a prognostic tumor marker. It has been shown that the high expression of NaPi2b may confer resistance to cancer chemotherapy, and may increase the metastatic potential in non-small cell lung cancer [10]. It was revealed that in lung cancer, *SLC34A2* induces resistance to crizotinib [18] when it undergoes molecular re-arrangement with *ROS-1*, a tyrosine kinase receptor. This re-arrangement creates a constitutively active tyrosine kinase that also increases the oncogenicity in lung cancer [19].

In order to consider *SLC34A2* as a potential prognostic and predictive marker of oncological diseases, we performed an analysis of the mutation and expression of *SLC34A2*, and assessed their effect on the life expectancy of patients from cancer studies which are publicly available online.

## 2. Materials and Methods

### 2.1. Data Collection and Preparation

The data were obtained from 4 open-access databases: cBioPortal, AACR Project Genie, The Insertional Cancer Genome Consortium (ICGC), and ArrayExpress (data downloaded on 21 December 2020). The mutational and the expression data were collected for the *SLC34A2* gene and mRNA. cBioPortal [20] was used to access the data from the 32 most recent TCGA (The Cancer Genome Atlas) studies. The TCGA data consisted of mutational, expression, and clinical data. From the AACR Project Genie database [21], 33 studies were utilized; only mutational data were collected. The ICGC data portal [22] was used to obtain 6 studies, and only mutational data were collected. From the ArrayExpress database [23], the E-MTAB-3732 study [24] was used to obtain expression data from tumor and relatively healthy samples divided into 24 groups. All of the collected studies and ArrayExpress samples were categorized by 27 tumor locations: adrenal gland, biliary tract, bladder, bone, bowel, breast, CNS and brain, cervix, esophagus and stomach, eye, head and neck, kidney, liver, lung, lymphoid, myeloid, ovary, pancreas, pleura, prostate, skin, soft tissue, testis, thymus, thyroid, uterus, and various tumors. The sample counts and study grouping are summarized in Supplementary Table S1. Different IDs other than the HGNC (HUGO Gene Nomenclature Committee) symbol (*SLC34A2*) were used: AFFY HG U133A 2 probe ID—204124_at, and Ensemble Protein ID—ENSP00000371483, TIGRFAMs ID—TIGR01013.

### 2.2. Prediction of the Functional Impact of Mutation

An analysis was conducted on the *SLC34A2* mutational data from 3 databases: cBioPortal, AACR Project Genie, and ICGC. Only missense and indel (insertion or deletion) *SLC34A2* missense and indel mutations were analyzed with tools for the prediction of the functional significance of mutations. The following tools were used to analyze missense mutations: PROVEAN [25], SIFT [26], PolyPhen-2 [27], Panther-PSEP [28], FATHMM [29], and Mutation Assessor [30]. In order to consider a missense mutation as pathogenic, it needed to be reported by at least five of the variant effect prediction tools. Indel mutations were analyzed with the PROVEAN tool. The determination of highly conserved regions of the *SLC34A2* protein was performed with the Conserved Domains and Protein Classification resource [31].

### 2.3. Analysis of the Expression Levels in Various Tumors

A comparison of the expression levels of *SLC34A2* in relatively healthy and tumor samples was carried out on the ArrayExpress *SLC34A2* expression data. A Wilcoxon test (*p* < 0.05) was performed in order to compare healthy and tumor samples.

### 2.4. Survival Analysis

A survival analysis was performed using the Kaplan–Meyer estimate (*p* < 0.05). For this analysis, only the cBioPortal TCGA studies were used. The tumor samples were divided into groups according to the level of *SLC34A2* mRNA expression (upregulation was considered as two standard deviations above the mean of the samples in each dataset) and by the presence or absence of an *SLC34A2* mutation.

For each study in which we found significant differences between the patient groups by the Kaplan–Meyer estimate, multivariate survival analysis was performed using Cox regression (*p* < 0.05). This analysis was carried out in order to determine whether the level of expression of *SLC34A2* or mutations in *SLC34A2* is one of the key factors linked to the survivability of patients. For this analysis, we used the clinical parameters which were available for each study.

## 3. Results

Mutational, expression and clinical data from 95 studies of oncological diseases were collected from four databases (cBioPortal, TCGA; cBioPortal, Genie; ICGC; and ArrayExpress). A total of 111283 samples was gathered and categorized by 27 tumor locations: adrenal gland (2 studies), biliary tract (1 study), bladder (3 studies), bone (2 studies), bowel (5 studies), breast (4 studies), CNS and brain (8 studies), cervix (2 studies), esophagus and stomach (9 studies), eye (1 studies), head and neck (5 studies), kidney (5 studies), liver (4 studies), lung (6 studies), lymphoid (5 studies), myeloid (4 studies), ovary (3 studies), pancreas (3 studies), pleura (2 studies), prostate (3 studies), skin (4 studies), soft tissue (3 studies), testis (1 studies), thymus (1 studies), thyroid (3 studies), uterus (4 studies), and various tumors (2 studies). The summary table can be found in Table 1, and the detailed information is located in Supplementary Table S1.

### 3.1. Evaluation of the Functional Impact of Missense and Indel Mutations in SLC34A2

Five hundred and twenty-one mutations in the *SLC34A2* gene were detected in the samples of three databases (cBioPortal, TCGA, *n* = 10,967; Genie, *n* = 85,369; ICGC, *n* = 2197): 441 missense mutations, 17 in-frame deletions, 14 out-of-frame mutations, three intergenic mutations, seven splice site mutations, seven nonsense mutations, four same-sense mutations, and 28 mutations of unknown origin.

**Figure 1:**
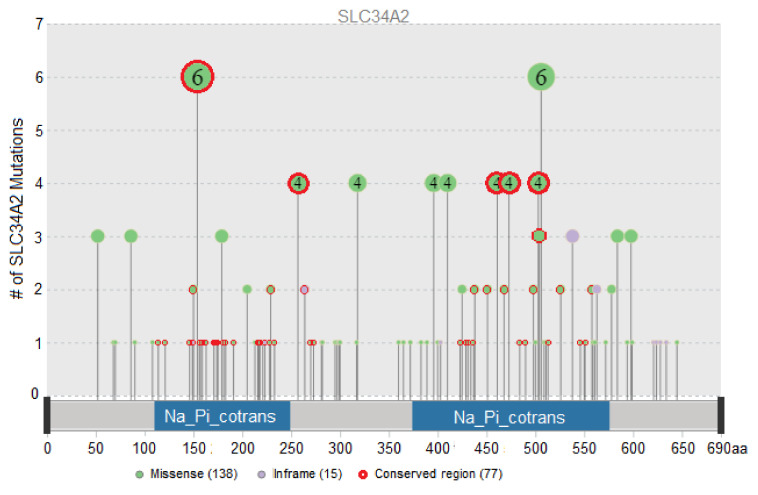
Functionally significant missense and indel mutations in the *SLC34A2* gene detected in the tumor samples of three databases (cBioPortal, TCGA, Genie, ICGC; *n* = 98,533). The x-axis shows the position of the amino acid in the *SLC34A2* protein sequence, the y-axis and the number of the dot represent the number of mutations found in the overall dataset, and the red outline of a dot indicates the conserved regions in the protein.

Out of the entire mutational dataset, 99 functionally significant missense mutations and 12 functionally significant insertion–deletion mutations in the *SLC34A2* gene were determined using tools for the prediction of the functional significance of mutations: PROVEAN, SIFT, PolyPhen-2, Panther-PSEP, FATHMM, and Mutation Assessor (Figure 1). In addition, three intergenic mutations were added to the list of functionally significant mutations. The list of functionally significant mutations, allele frequencies, rsIDs (identification number from dbSNP database), tumor locations, sample counts, and the indication of highly conserved regions are stated in Supplementary Table S2.

The most frequent mutations by the sample count in total (the numbers are given in total for all studies, *n* = 98,533) are intergenic mutation *SLC34A2-ROS1* (10 samples), which was found only in lung cancer samples; p.T154A (six samples); p.P506S/R/L (six samples); p.G257A/E/R (four samples); p.S318W (four samples); p.A396T (four samples); p.P410L/S/H (four samples); p.S461C (four samples); p.A473T/V (four samples); and p.Y503H/C/F (four samples). The sample counts according to the tumor locations are displayed in Table 2.

Twenty-one functionally significant mutations in the *SLC34A2* gene were found in two or more tumor locations (Supplementary Table S3). Several mutations were found in more than three locations: p.T154A (breast, esophagus and stomach, lymphoid, ovary, pleura), p.A396T (lung, thymus, uterus), p.A473T (esophagus and stomach, head and neck, lung), and p.F538del (lung, lymphoid, kidney). The most frequently occurring tumor locations (with the highest number of functionally significant mutations in the *SLC34A2* gene) were the skin, lung, bowel, and uterus, with 23, 22, 17, and 17 unique mutations respectively (Supplementary Table S4).

### 3.2. Comparison of the Expression Levels of SLC34A2 between Relatively Healthy and Tumor Tissues

The expression data used for comparison between relatively healthy and tumor tissue samples were gathered from the ArrayExpress E-MTAB-3732 study (*n* = 12,750). It should be mentioned that the data we used did not contain methylation information that could influence the expression of *SLC34A2*, and we have not taken this factor into account in our analysis.

**Figure 2:**
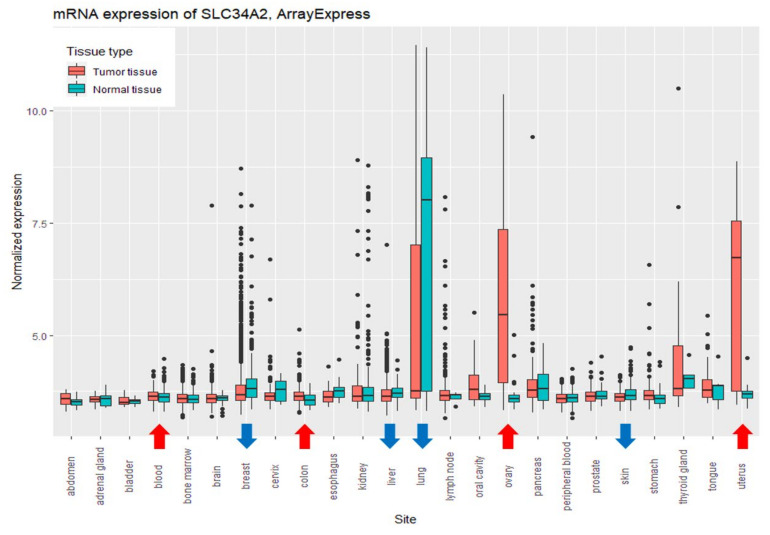
Comparison of the mRNA expression levels of *SLC34A2* between relatively healthy and tumor tissues (ArrayExpress: *n* = 12,873; Wilcoxon test, *p* < 0.05).

Comparing the levels of *SLC34A2* expression between relatively healthy and tumor tissues (Wilcoxon test, *p* < 0.05), the higher levels of expression were found in tumor tissues of the myeloid, bowel, ovary, and uterus, and the lower levels were found in samples of breast, liver, lung, and skin tumors (Figure 2).

### 3.3. Characterization of the SLC34A2 Gene as a Prognostic Marker

A survival analysis was conducted on 32 cBioportal TCGA studies using the Kaplan-Meier estimator. The tumor samples were divided into groups in two ways: by the level of *SLC34A2* mRNA expression and by the presence or absence of an *SLC34A2* mutation. It must be mentioned that not all of the cBioportal TCGA samples had lifespan information; thus, the datasets may be smaller (Supplementary Table S1).

**Figure 3:**
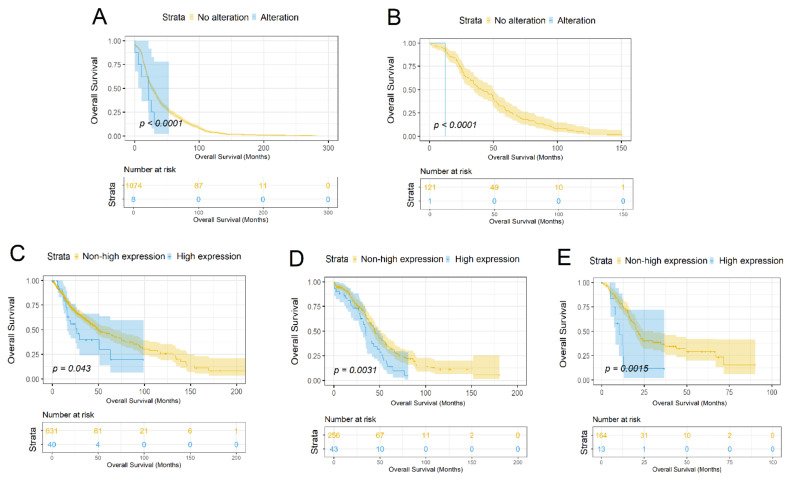
Survival analysis based on mutational and expression profiles of *SLC34A2* (cBioPortal, TCGA; Kaplan–Meier estimator, *p* < 0.05). The tumor samples were divided into groups according to the presence or absence of *SLC34A2* mutation: (**A**) breast: breast invasive carcinoma, *n* = 1082; (**B**) thymus: thymoma, *n* = 122. The tumor samples were divided into groups according to the level of *SLC34A2* mRNA expression: (**C**) CNS and brain: brain lower grade glioma, *n* = 514; glioblastoma multiforme, *n* = 592; (**D**) ovary: ovarian serous cystadenocarcinoma, *n* = 299; (**E**) pancreas: pancreatic adenocarcinoma, *n* = 179).

The life expectancy of patients with mutations in the *SLC34A2* gene is significantly lower than in patients without gene alterations in studies of breast (*p* < 0.042) and thymus (*p* < 0.0008) tumors (cBioPortal, TCGA: Breast invasive carcinoma, *n* = 1082, Thymoma, *n* = 122; Figure 3A-B).

The life expectancy of patients with a high expression of *SLC34A2* is significantly lower than that of patients with a lower expression of the gene in studies of brain (*p* < 0.043), ovarian (*p* < 0.0031), and pancreatic (*p* < 0.0015) cancer (cBioPortal, TCGA: Brain Lower Grade Glioma, *n* = 514, Glioblastoma multiforme, *n* = 592, Ovarian serous cystadenocarcinoma—*n* = 299, Pancreatic adenocarcinoma, *n* = 179, *p* < 0.05; Figure 3C–E).

Multivariate survival analysis was performed using Cox regression (*p* < 0.05) for each dataset in which we found significant differences between the patient groups according to the Kaplan–Meyer estimate.

For the breast cancer dataset, the results of the multivariate survival analysis showed that the hazard ratio for the group with a mutation of *SLC34A2* gene is 14.42 (CI: 4.24–49.05; Supplementary Figure S1A); the parameters with a higher hazard ratio were Neoplasm Disease Lymph Node Stage N3, Neoplasm Disease Stage III and IV. The result for the thymoma dataset indicated as significant only the presence of the mutation of *SLC34A2* (HR = 24.57, CI: 1.87–323.65; Supplementary Figure S1B). The multivariate analysis of the CNS and brain cancer dataset showed that the level of *SLC34A2* mRNA expression has a hazard ratio of 2.07 (CI: 0.99–4.3; Supplementary Figure S1C); the parameter with a higher hazard ratio was only Neoplasm Disease Lymph Node Stage N3. For the ovary and pancreas datasets, the level of *SLC34A2* expression had the highest hazard ratios—1.77 (CI: 1.19–2.63; Supplementary Figure S1D) and 3.51 (CI: 1.6–7.71; Supplementary Figure S1E) respectively—and for the pancreas dataset, the level of expression was the only significant parameter in the analysis.

## 4. Discussion

In order to assess the possible role of *SLC34A2*as a prognostic marker of cancer, we performed the analysis of the *SLC34A2* mutational data, the *SLC34A2* mRNA expression data, and the survival data of cancer patients which were publicly available online.

The analysis of mutations in the *SLC34A2* gene revealed that most of them are associated with skin, lung, bowel, and uterine cancer types. The most frequent functionally significant mutation is the intergenic mutation *SLC34A2-ROS1*, which was found only in lung cancer samples, but in other studies, this mutation was connected not only with lung cancer [19] but also with bowel [32], stomach [33], and ovary [34] cancers. We found no association between intergenic mutation *SLC34A2-ROS1* and the survivability of lung cancer patients in our study. Among the functionally significant single nucleotide polymorphisms, several frequent mutations should be mentioned: p.T154A, p.P506S/R/L, p.G257A/E/R, p.S318W, p.A396T, p.P410L/S/H, p.S461C, p.A473T/V, and p.Y503H/C/F.

The comparisons of *SLC34A2* expression between healthy and tumor tissues showed a distinction in their expression level: upregulation was found in samples of myeloid, bowel, ovarian, and uterine tumors; downregulation was found in tumor samples of breast, liver, lung, and skin cancer tumors. Our results are in accordance with the published data. Upregulation in myeloid and bowel tumors was found in several studies [35,36]; the overexpression of *SLC34A2* was found in endometrioid and papillary serous ovarian carcinomas [14,15,16,37,38], and it was shown that in uterine (endometrial) cancer, *SLC34A2* expression appeared relatively increased [14,16]. Downregulation in breast [39], liver [40], and lung [7,8,9] tumors was discovered in several studies. It should be noted that the research also shows the upregulation of *SLC34A2* in lung tumor samples [7,41]. Our analysis for the first time showed that the expression of *SLC34A2* is downregulated in skin cancer.

We conducted a survival analysis of cancer patients, taking into account the obtained mutational data and the expression profile of *SLC34A2*. The analysis of the mutational data showed the lower life expectancy of patients with *SLC34A2* alterations in the studies of breast and thymus cancers. The results of the multivariate survival analysis showed that the mutational profile of *SLC34A2* plays a significant role in the survivability of breast and thymus cancer patients. Both studies contained the discovered functionally significant mutations. Breast samples contained the following functionally significant mutations: p.D68G and p.P504L (the latter is located in a conserved region). These alterations could be a reason for poor prognosis. It was mentioned that *SLC34A2* could influence chemotherapy [42] and could establish reasoning for a targeting pathway [43], and the downregulation of *SLC34A2* could play a role in breast cancer progression [39]. In thymus samples, we found one functionally significant mutation: p.A396T. Our research, for the first time, showed the correlation between patient survivability and *SLC34A2* alterations in thymus cancer.

The analysis of the *SLC34A2* expression data showed the potential impact of *SLC34A2* upregulation being related to poor survival prognosis in ovarian, pancreatic, and brain tumors. The multivariate survival analysis demonstrated that the level of *SLC34A2* expression is one of the key parameters for ovarian, pancreatic, and brain cancer patient survival. We confirmed the published data that *SLC34A2* is upregulated in ovarian tumors compared to relatively healthy ovarian tissues [37]. Besides this, we showed that patients with the higher expression of *SLC34A2* had a lower life expectancy. Interestingly, we have shown previously that *SLC34A2* is overexpressed in well-differentiated papillary serous and endometrioid ovarian carcinomas which usually have a good prognosis [44]. This inconsistency may be related to the molecular heterogeneity of ovarian tumors, and it requires further consideration. The expression of *SLC34A2* in pancreatic cancer has not yet been researched, and our study for the first time showed the correlation between patient survivability and *SLC34A2* expression. Concerning brain cancer, it has been shown previously that *SLC34A2* is overexpressed in glioma [45], but the relation of increased expression with the life expectancy of patients has not been studied.

In conclusion, we analyzed a significantly large dataset of the *SLC34A2* mutational data, the *SLC34A2* expression data, and the survival data of cancer patients (*n* = 111,283). One hundred and eleven functionally significant mutations were discovered, and it was found that functionally significant mutations of *SLC34A2* might be involved in the reduction of the life expectancy of breast (p.D68G, p.P504L) and thymus (p.A396T) cancer patients. Thus, for these types of oncological diseases, the mutational profile of *SLC34A2* can be a potential prognostic marker. It was revealed that *SLC34A2* mRNA overexpression decreases the lifespan of patients with brain, ovarian, and pancreatic tumors; therefore, the upregulation of *SLC34A2* can be a prognostic marker for these tumors. Furthermore, we suggest that *SLC34A2* upregulation—not only for ovary but also for myeloid, bowel, and uterine tumors—can be considered as a potential predictive marker for targeted therapy with monoclonal antibodies, including XMT-1536 and XMT-1592. Taking into account the molecular heterogeneity of tumors, we concluded that the obtained data require further investigation considering the molecular subtypes of these tumors.

## Figures and Tables

**Table 1:** Summary of the collected data representing the number of samples for every tumor location and every database.

Location	cBioPortal, TCGA	ICGC	cBioPortal, Genie	ArrayExpress
Adrenal gland	92	-	-	36
Biliary Tract	36	-	-	-
Bladder	411	-	2118	20
Bone	-	-	718	1987
Bowel	594	411	9682	947
Breast	1084	569	11,742	1505
CNS and Brain	1106	-	6609	791
Cervix	297	-	-	292
Esophagus and Stomach	622	-	4419	259
Eye	80	-	-	-
Head and neck	523	-	2223	120
Kidney	860	-	1665	293
Liver	372	773	-	636
Lung	1053	170	14,844	794
Lymphoid	48	274	2625	565
Myeloid	200	-	2820	2431
Ovary	585	-	3527	573
Pancreas	184	-	3613	235
Pleura	87	-	620	-
Prostate	494	-	3490	314
Skin	448	-	4537	647
Soft Tissue	433	-	2752	-
Testis	149	-	-	-
Thymus	123	-	-	-
Thyroid	500	-	1398	30
Uterus	586	-	2905	275
Various tumors	-	-	3065	-
Sample Count	10,967	2197	85,369	12,750

**Table 2:** The most frequent mutations in *SLC34A2* with the sample count according to the tumor location and total sample count, and the indication of highly conserved regions.

Protein Consequence	Tumor Locations and Sample Count	Total Sample Count	Conserved Region
*SLC34A2-ROS1*	Lung (10)	10	
p.T154A	Breast (1), Esophagus and Stomach (2), Lymphoid (1), Ovary (1), Pleura (1)	6	+
p.P506S/R/L	Bowel (1), Liver (2), Lung (1), Skin (2)	6	
p.G257A/E/R	Lung (1), Ovary (1), Skin (2)	4	+
p.S318W	Breast (2), Kidney (2)	4	
p.A396T	Lung (1), Thymus (1), Uterus (2)	4	
p.P410L/S/H	Skin (3), Uterus (1)	4	
p.S461C	Lung (4)	4	+
p.A473T/V	Esophagus and Stomach (1), Head and neck (1), Lung (1), Uterus (1)	4	+
p.Y503H/C/F	Bowel (2), Esophagus and Stomach (1), Head and neck (1)	4	+

## Data Availability

Publicly available datasets were analyzed in this study. These datasets can be found here (the data was downloaded on 21 December 2020): http://www.cbioportal.org, https://genie.cbioportal.org/, https://dcc.icgc.org/, and https://www.ebi.ac.uk/arrayexpress/experiments/E-MTAB-3732.

## Supplementary Materials

The following are available online at https://www.mdpi.com/article/10.3390/biom11121878/s1. Table S1: Overview of the collected data—95 studies across 4 databases (cBioPortal, TCGA; cBioPortal, Genie; ICGC; ArrayExpress). Table S2: List of functionally significant missense and indel mutations in *SLC34A2* gene. Table S3: Functionally significant mutations in the *SLC34A2* gene found in two or more tumor locations. Table S4: Tumor locations with the highest numbers of functionally significant mutations in the *SLC34A2* gene. Figure S1: Multivariate survival analysis using clinical parameters and the *SLC34A2* group.
